# Pigments of aminophenoxazinones and viridomycins produced by termite-associated *Streptomyces tanashiensis* BYF-112

**DOI:** 10.3389/fmicb.2022.1110811

**Published:** 2023-01-16

**Authors:** Shuxiang Zhang, Jun Wu, Zhou Jiang, Le Zhang, Tao Song, Xinhua Liu, Caiping Yin, Yinglao Zhang

**Affiliations:** ^1^School of Life Sciences, Anhui Agricultural University, Hefei, China; ^2^School of Pharmacy, Anhui Medical University, Hefei, China

**Keywords:** *Streptomyces tanashiensis* BYF-112, natural pigments, aminophenoxazinones, viridomycins, cytotoxic and antibacterial activities

## Abstract

Termite-associated *Streptomyces tanashiensis* BYF-112 was found as a potential source for yellow and green pigments, which were stable under the tested temperature, light and metal ions. Eight metabolites (**1**–**8**), including four new natural yellow pigments aminophenoxazinones (**1**–**4**), and two rarely iron dependent green pigments viridomycin A and F (**9**–**10**) were isolated from BYF-112 cultured in YMS and YMS treated with FeSO_4_, respectively. The metabolites **2**–**4** displayed a significant safety performance on the normal liver cell line L-02, while the metabolite **1** showed weak cytotoxicity against the L-02 and several cancer cells. Especially, in the filter paper disc tests, the compound **1** possessed strong antibacterial activity against methicillin-resistant *Staphylococcus aureus* (MRSA) with the zone of inhibition (ZOI) of 15.3 mm, which was equal to that of referenced levofloxacin (ZOI = 15.2 mm). And the metabolite **1** also showed moderate antibacterial activities against *Micrococcus teragenus* and *S. aureus*, with the ZOI values of 15.3 and 17.2 mm. In addition, by the minimum inhibitory concentration (MIC) assay, the compound 1 displayed potential antibacterial activities against *M. teragenus*, *S. aureus* and MRSA, with the MIC values of 12.5, 12.5, and 25.0 μg/ml, respectively. The present results indicate that BYF-112 may be a promising source for safe and bioactive pigments, which can be used for further development and industrial applications.

## 1. Introduction

Edible pigments play a very important role in food qualities and consumer acceptance of products. With the increasing demand for pigments production in the world, the market of food colorants was estimated to reach USD 5.12 billion by 2023 with a five-year compound growth rate of 5.7% (Rana et al., 2021). Usually, food colors are categorized into natural and synthetic colors based on the origin (Teixeira et al., 2022). Synthetic colors have been widely used in the food industry for many years, but they pose serious health risks (Saini et al., 2018). Consumers are demanding natural pigments derived from animals, plants, insects and microbes for their safe, non-toxic, non-carcinogenic and biodegradable characteristic (Hosseini et al., 2021). Among these sources, natural edible colors by microbial fermentation have gained significant attention due to the abundance of raw materials without dependence on seasonal variations, cost effective production, and higher concentration with easier purification of products (Panesar et al., 2015; Pombeiro-Sponchiado et al., 2017). To satisfy these requirements, investigations on natural pigments, especially those derived from microorganisms, have increased (Chatragadda et al., 2019a; Chatragadda and Dufosse, 2021; Ramesh et al., 2022).

And several kinds of pigments derived from microorganisms have been developed as industry products, including prodigiosin (Ramesh et al., 2020), phycocyanin (Ashaolu et al., 2021), ankaflavin (Hsu et al., 2011; Dufossé, 2017), astaxanthin (Patel et al., 2022), *β*-carotene (Lopes et al., 2009; Dufossé, 2017), monascorubramin (Dufossé, 2018), riboflavin (De Faria et al., 2022), rubropunctatin (Zheng et al., 2010), anthraquinones (Venil and Lakshmanaperumalsamy, 2009). These food grade pigments obtained from microbial sources can also have health benefits as anti-cancer, anti-microbial and antioxidant, etc. (Rao et al., 2017).

Aminophenoxazinones are highly colored dyes, possessed a number of promising properties like anticarcinogenic, antifungal, antiparasitic, antibacterial or antimicrobial activities (Zorrilla et al., 2021) which consist of tricyclic structures with double bonds in aromatic systems containing oxygen and nitrogen atom (Pasceri et al., 2013). Thus, aminophenoxazinones are good candidate food grade pigments. Here we obtained four new natural aminophenoxazinones pigments **1**–**4**, two rarely green pigments (**9**–**10**) from *Streptomyces tanashiensis* BYF-112 associated with *Odontotermes formosanus* for the first time. In this context, the objective of the work was to evaluate the potential of the production of natural pigments by insect associated actinomycete isolated from *O. formosanus*, that is (i) characterize the pigment by UV–Vis spectroscopy and evaluate its stability in relation to temperature, light and metal ions, (ii) separate and identify the pigments by column chromatography, electrospray ionization mass spectroscopy and NMR; and (iii) evaluate the cytotoxic and antimicrobial activities of the pigments.

## 2. Materials and methods

### 2.1. Influence of iron on the metabolite of BYF-112

*Streptomyces tanashiensis* BYF-112 was isolated from the workers of *O. formosanus* as described in our previous work and was preserved on YMS (1.0 g L^–1^ KNO3, 1.5 g L^–1^ yeast extract, 10 g L^–1^ soluble starch, and 20 g^–1^ agar) slants at 4°C until use (Long et al., 2022). From a 7-day old YMS solid culture plate, a conidial spore suspension was prepared by adding 15 ml of distilled water containing 0.2% Tween 80 (*w*/*v*) under agitation by glass rod. To study the effect of iron, aliquots (2 ml) of spore suspension were added into 100 ml of YMS production media supplemented with deionized water dissolved FeSO_4_•7H_2_O at the concentrations of 50 mg/l. Equal amount of deionized water was added to the control group. The filter of each fermentation broth was extracted three times with equal amount of ethyl acetate (EtOAc) after fermenting at 28°C in a shaker rotating at 180 rpm for 7 days. The EtOAc extracts were fast analyzed by thin-layer chromatography (TLC).

### 2.2. Pigments stability

Solution (250 μg/ml) of yellow pigments (YP) and the green pigments (GP) were dissolved in DMSO and deionized water respectively, and stored in the dark. The pigments stability under heat, light and metal ions were performed by the methods described in detail previously with slight modification (Zhu et al., 2020; Wu et al., 2022). Heat stability: the tested tubes were placed into 101-3A electric thermostatic drying oven (Changzhou, Jiangsu, China) at 40, 60, 80, 100, and 120°C for 2 h. The samples were then removed and quickly cooled to room temperature in an ice bath for further analysis. Stability under condition with light and dark: the effect of light on pigment stability was determined by after exposure to indoor incandescent light and stored at dark place for 1–7 days. Stability under condition with metal ions: the effect of metal ions (Ca^2+^, Zn^2+^, Mg^2+^, Na^+^, and K^+^, 1 mmol/l) on the pigment was determined in the dark for 2 days.

The retention rate of YP and GY were calculated at the maximum absorbance 407 and 700 nm respectively, using UV–Visible spectrophotometer (UNICO UV-2800) according to following formula (Wu et al., 2022).

Retention rate of YP/GP = *A*1/*A*0 × 100%.

*A*1/*A*0: the absorbance of YP/GP after/before treatment.

### 2.3. Fermentation

The fermentation procedure was described as the previously reference with slight modifications (Zhang et al., 2020). The fresh spores of *S. tanashiensis* BYF-112 cultivated on YMS agar plates at 28°C for 3–5 days were inoculated into 250 ml Erlenmeyer flask containing 100 ml YMS liquid culture medium (10 g soluble starch, 1.5 g yeast extract, 1.0 g KNO_3_, 1,000 ml deionized water). After 3 days of incubation at 28°C on rotary shakers at 180 rpm, 30 ml of cultured liquid was transferred as a seed into Erlenmeyer flasks (1,000 ml), each containing 400 ml YMS broth (or YMS broth supplemented with FeSO_4_•7H_2_O). The cultures were continuously shaken at 180 rpm for 7 days at 28°C to produce pigments.

### 2.4. Extraction, isolation, and identification of metabolites

The yellow culture broth (16 l) of BYF-112 grown on a YMS liquid medium was filtered by gauze to obtain the supernatant, which was then extracted with ethyl acetate (EtOAc, 3 × 16 l) at room temperature. The EtOAc phase was evaporated *in vacuo* to afford crude extract (6.6 g). The crude extract was subjected to a silica gel column eluting with a stepwise gradient of CH_2_Cl_2_/MeOH (100, 0–100:16, *v*/*v*) to give five fractions (Fr1–Fr5). Compound **6** (15 mg) was crystallized from the CH_2_Cl_2_ solution from the Fr2 (CH_2_Cl_2_/MeOH, 100:1, *v*/*v*). Fr2 was repeatedly chromatographed over a Sephadex LH-20 column (MeOH) to yield metabolite **5** (6.0 mg). Fr3 (CH_2_Cl_2_/MeOH, 100:2, *v*/*v*) was further fractionated on a silica gel column, eluting with (CH_2_Cl_2_/MeOH, 100:0, 100:1 and 100:2, *v*/*v*) to give three subfractions (R1–R3). Subfractions R2 and R3 were loaded onto a Sephadex LH-20 column eluting with MeOH to give compounds **1** (40.2 mg) and **3** (5.2 mg), respectively. Compound **2** (30.1 mg) was crystallized from the methanol-dichloromethane binary solvent solution of Fr4 (CH_2_Cl_2_/MeOH, 100:4). The remaining part of Fr4 was further purified by a Sephadex LH-20 eluting with MeOH to yield compounds **7** (8.8 mg) and **8** (7.9 mg). Fr5 (CH_2_Cl_2_/MeOH, 100:16, *v*/*v*) was also loaded onto a Sephadex LH-20 column to yield compound **4** (8.6 mg).

The green culture broth (8 l) of BYF112 grown on a YMS liquid media supplemented with FeSO_4_•7H_2_O was centrifuged to afford the liquid phase at 6,000 rpm for 10 min. The liquid phase was passed through a macroporous resin column D-101 (Shangxi Lebobiochem Co., Ltd., China), rinsed with deionized water until the color of the effluent turned colorless, and then eluted with methanol. The organic fraction was evaporated *in vacuo* to afford crude extract (2.6 g). The crude extract was subjected to a silica gel column eluting with a stepwise gradient of CH_2_Cl_2_/MeOH (100:4, 100:8, and 100:16, *v*/*v*) to give two fractions (Fr1–Fr2). Fractions Fr1 and Fr2 were further purified by a Sephadex LH-20 eluting with MeOH to yield compounds **9** (20.1 mg) and **10** (10.9 mg), respectively.

Structural identifications of the metabolites were made on the basis of the spectroscopic analysis. NMR spectra were recorded on an Agilent II DD2 instrument operating at 600 MHz for ^1^H and 150 MHz for ^13^C, while 2D spectra (COSY, HMQC, HMBC, and DEPT) were obtained using standard Agilent software. Chemical shifts were given in parts per million (*δ*) downfield from the TMS internal standard. A Mariner Mass 5,304 instrument was used to measure the HR-ESI-MS spectra.

### 2.5. Cytotoxicity assay

The metabolites **1**–**4** were evaluated for their cytotoxic activities against human malignant melanoma cell line (A375), human ovarian cancer cell line (SKOV-3), human breast cancer cell line (MDA-MB231), human gastric cancer cell line (MGC-803) and human normal cell line (L-02) with the MTT method (Wang et al., 2022). The cell lines were maintained in Dulbecco’s modified eagle medium (DMEM) supplemented with 10% fetal bovine serum (FBS) along with 1% penicillin and streptomycin. Cells were grown at 37°C in an atmosphere with 5% CO_2_ and then seeded in 96-well plates and permitted to grow for 20 h. Then tested compounds at preset concentrations were added. The 3-(4,5-dimethylthiazol-2-yl)-2,5-diphenyltetrazolium bromide (MTT; 20 μl, 5 mg/ml) was added into each well after a 48 h exposure period. After an additional 4 h further incubation, the medium was replaced by DMSO (150 μl), then shaking the plates for 15 min. The absorbance at 490 nm were recorded. The percentage of cell viability was calculated using the following formula: Cell viability (%) = the OD of tested group/the OD of CK × 100%. Finally, IC_50_ values were determined by EXCEL. The azithromycin was used as positive control.

### 2.6. Antimicrobial activity

The *Staphylococcus aureus* (ATCC6538), methicillin-resistant *S. aureus* (MRSA, SA2), *Escherichia coli* (ATCC8739) and *Micrococcus tetragenus* (ATCC35098) donated by Professor Ting Xue from Anhui Agricultural university were used as a target pathogen for the examination of antibacterial activity of the metabolites. The disc diffusion (Özogul et al., 2022) and minimum inhibitory concentrations (MICs; Cui et al., 2022) methods were performed to evaluate the antibacterial activities of the new natural metabolites **1**, **2**, and **4**. Filter paper disks (6 mm) with metabolite dissolved in DMSO in a concentration (30 μg/filter paper) were added to the culture medium, and the plates were incubated at 37°C for 12 h. Filter papers with DMSO and levofloxacin were set as negative and positive controls, respectively. All experiments were performed in triplicate, and data were shown as mean values standard deviation. And the MICs of the compounds were determined by the method as we previously described (Cui et al., 2022).

## 3. Results and discussion

### 3.1. Effect of iron on the metabolic profile of *Streptomyces tanashiensis* BYF-112

Effect of Iron on the metabolic profile of *S. tanashiensis* BYF-112 was displayed in Supplementary Figure S1A. The result showed that the color of the fermentation broth exhibited a great difference between YMS and YMS culture broth treated with iron, which presented yellow and green, respectively. Furthermore, TLC profiles indicated that two green spots were observed in iron treated extract, and only yellow spots were seen in the extracts of iron free cultures (Supplementary Figure S1B). These results showed that iron had a great influence on the metabolite profiles of the strain BYF-112.

### 3.2. Stability of yellow and green pigments

Previous reports showed that the color stability of natural pigment was affected by external factors such as heat, light, and metal ions (Zhang et al., 2006; Zhu et al., 2020). Therefore, the stability of YP and GP under different environmental conditions (heat, light, and metal ions) were evaluated in this study.

**Figure 1:**
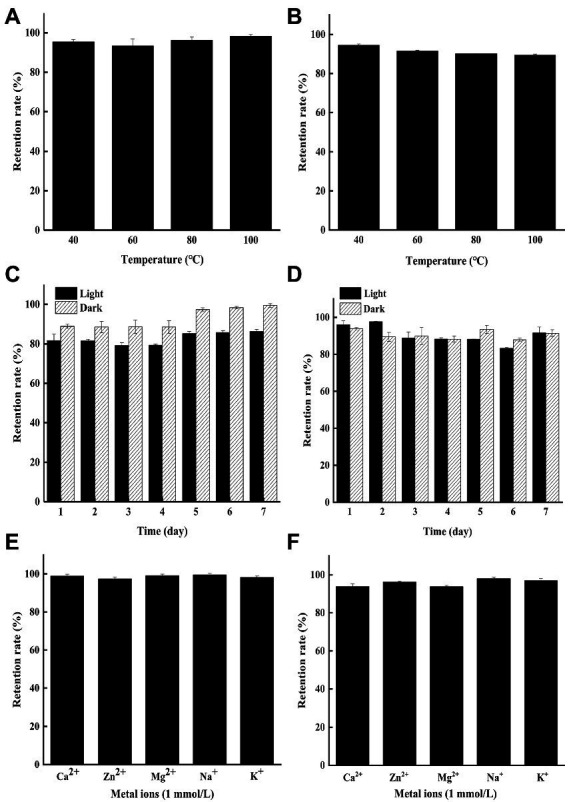
The stability of the yellow pigments (YP) and green pigments (GP) in different tested conditions. **(A)** Effects of heat on the stability of the YP. **(B)** Effects of heat on the stability of the GP. **(C)** Effects of light on the stability of the YP. **(D)** Effects of light on the stability of the GP. **(E)** Effects of metal ions on the stability of the YP. **(F)** Effects of metal ions on the stability of the GP.

As shown in Figures 1A,B, both YP and GP presented strong heat stability with the residual rate of 89.44–98.22% under exposure to 40–100°C for 2 h at the concentration of 250 μg/ml. The result indicated that microbial pigments might be applied in the food industry due to their superior thermal stability, compared to other natural pigments (Vendruscolo et al., 2013; Teran Hilares et al., 2018).

The color stability of YP and GP stored in light and dark was conducted under being treated for 1–7 days at 25°C (Figures 1C,D). The results showed that the YP retention rate (86.23%) in the indoor incandescent light decreased slightly, compared to that of in the dark for 7 days (99.44%), while the GP remained stable under light and dark for 7d with the retention rate of 91.57 and 91.42%, respectively. Hence, both pigments can be preserved in light to some extent.

The effect of metal ions (Ca^2+^, Zn^2+^, Mg^2+^, Na^+^, K^+^, 1 mmol/l) on the pigment was determined in the dark for 2 days. The results showed that both YP (Figure 1E) and GP (Figure 1F) were stable with the retention rate of more than 92% under the treatments of different metal ions.

### 3.3. Identification of the secondary metabolites from *Streptomyces tanashiensis* BYF-112

**Figure 2:**
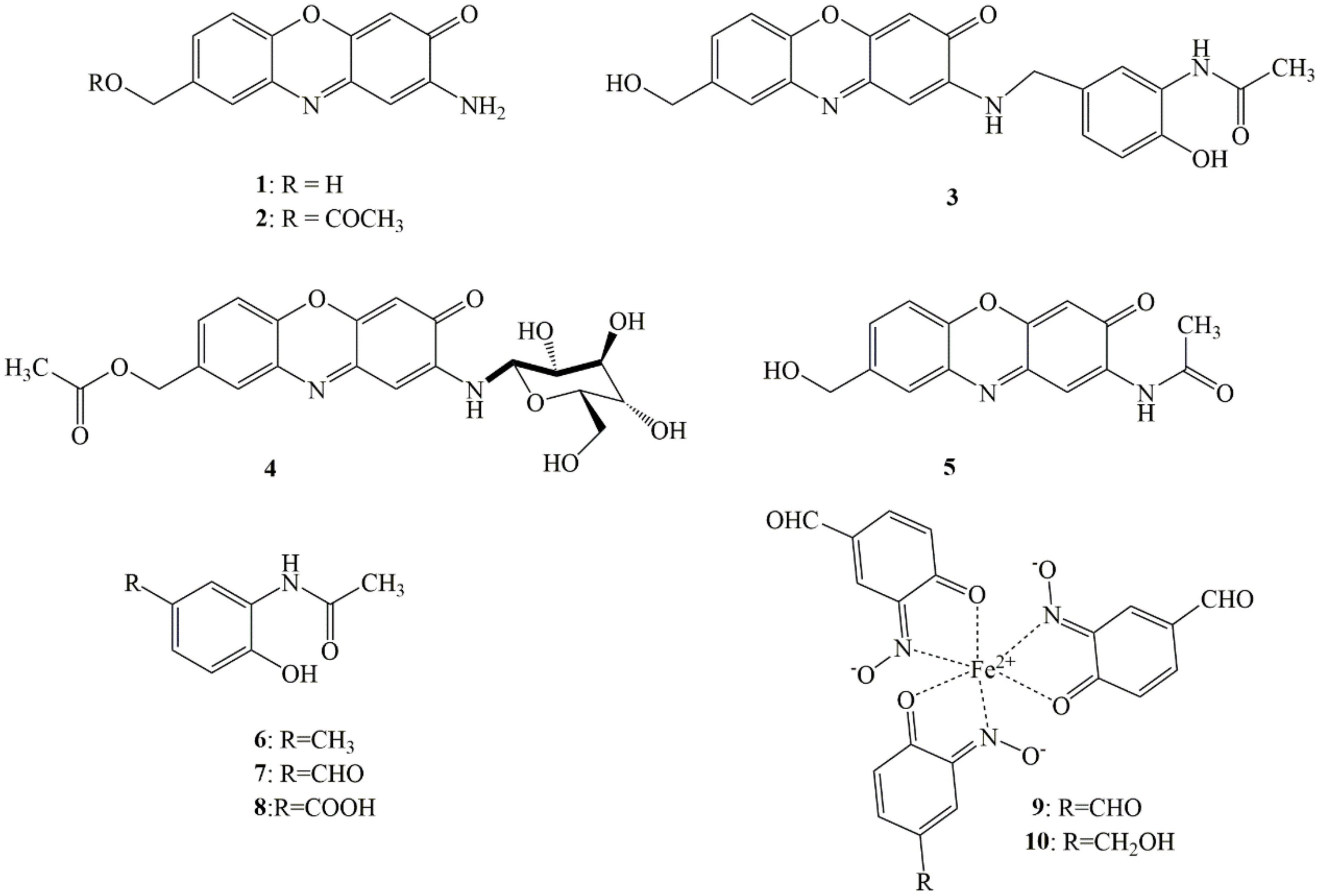
Chemical structures of metabolites **1**–**10** of *Streptomyces tanashiensis* BYF-112.

Four new natural compounds **1**–**4**, along with six known metabolites **5–10** were isolated from the culture of *S. tanashiensis* BYF-112. Chemical structures of compounds **1**–**10** are shown in Figure 2.

Compound **1** was obtained as a yellow brown powder, and its molecular formula C_13_H_10_N_2_O_3_ was deduced from HR-ESI-MS data (*m/z* [M + H]^+^ 243.0757, which was quite in accordance with ^1^H and ^13^C NMR data (Supplementary Figures S2–S4). ^1^H NMR and ^13^C NMR data were as following: ^1^H NMR (DMSO-*d*_6_, 600 MHz) *δ*_H_ 4.55 (2H, d, *J* = 5.6 Hz), 5.30 (1H, m), 6.33 (H, s), 6.33 (H, s), 6.74 (2H, brs), 7.38 (1H, d, *J* = 8.3 Hz), 7.43 (1H, d, *J* = 8.3 Hz), 7.60 (1H, brs); ^13^C NMR (DMSO-*d*_6_, 150 MHz) *δ*_C_ 62.5 (CH_2_), 98.8 (CH), 103.6 (CH), 115.9 (CH), 125.7 (CH), 127.5 (CH), 133.8 (C), 140.2 (C), 141.0 (C), 147.6 (C), 148.5 (C), 149.2 (C), 180.6 (C). The data were almost no different from 2-amino-8-hydroxymethyl-3H-phenoxazin-3-one, a semisynthetic derivative of the pigment aminophenoxazinone described in the literature (Pasceri et al., 2013). However, to our knowledge, the compound was first discovered as a new natural product.

Compound **2** was purified as a yellow brown powder, and its molecular formula C_15_H_12_N_2_O_4_ was deduced from HR-ESI-MS ion peak at m/z [M + H]^+^ 285.0860, which was consistent with ^1^H and ^13^C NMR data (Table 1; Supplementary Figures S5–S11). The ^1^H NMR and ^13^C NMR spectra of **2** were resembled to those of **1,** except an acetyl unit signal (*δ*_C_ 170.1, 20.6; *δ*_H_ 2.09, s, 3H) at position 12 in **1** appeared to be COCH_3_, which corresponded to the increase in molecular of compound **2** by 42 amu compared to **1**. Further structure elucidation of **2** was established by the 1D and 2D NMR spectra. The ^1^H NMR data exhibited the protons resonances of a trisubstituted aromatic ring proton signals at *δ*_H_ 7.49, (1H, d, *J* = 8.4 Hz, H-6), *δ*_H_ 7.44, (1H, d, *J* = 9.5 Hz, H-7) and *δ*_H_ 7.69, (1H, s H-9), another two aromatic protons at *δ*_H_ 6.34 (1H, s, H-1), 6.36 (1H, s, H-4), one oxygenated methylene proton peak at *δ*_H_ 5.16 (2H, s, H-11), one methyl protons of acetyl group resonances line at *δ*_H_ (2.09, 3H, s, H-14) and one α-amino proton signal at *δ*_H_ 6.81 (2H, d, *J* = 6.7 Hz, NH_2_-2). The observation agreed with its ^13^C NMR spectra, which displayed as anticipated one methyl carbon (*δ*_C_ 20.6, CH_3_, C-14), one methylene carbon (*δ*_C_ 64.6, CH_2_, C-11), one carbonyl carbon (*δ*_C_ 180.1, CO, C-2), and 11 aromatic carbon resonance lines (*δ*_C_ 98.4, 142.4, 103.4, 148.8, 141.4, 115.9, 128.3, 133.3, 127.2, 133.4, and 148.4). These substructures were pieced together by the 2D NMR experiments including its ^1^H–^1^H COSY spectrum highlighting the two coupling sequences (11-H to 7-H and 9-H), and its HMBC spectrum (Supplementary Figure S12) indicating the key correlations of H-1 to C-3 and C-4a, and of H-4 to C-2 and C-10a, and of H-6 to C-5a, C-8 and C-9a, and of H-7 to C-11 and C-5a, and of H-9 to C-5a and C-11, and of H-8 to C-8 and C-9, and of 2-NH_2_ to C-1 and C-3. Thus, the structure of metabolite **2** was determined as a new aminophenoxazinone derivative and named exfoliazone B.

**Table 1:** ^1^H and ^13^C NMR data for compounds **2** and **3** in DMSO-*d*_6_.

No.	**2**		**3**	
	*δ*_H_, mult (*J* in Hz)	*δ*_C_	*δ*_H_, mult (*J* in Hz)	*δ*_C_
1	6.36, s	98.4, CH	6.04, s	96.3, CH
2		142.4, C		145.7, C
3		180.1, C		179.9, C
4	6.36, s	103.4, CH	6.36, s	103.3, CH
4a		148.8, C		149.3, C
5a		141.4, C		140.7, C
6	7.49, d, 8.0	115.9, CH	7.45, d, 8.0	115.6, CH
7	7.44, d, 9.5	128.3, CH	7.38, d, 8.0	127.2, CH
8		133.3, C		140.0, C
9	7.69, brs	127.2, CH	7.58, s	125.3, CH
9a		133.4, C		133.5, C
10a		148.4, C		149.9, C
11	5.16, s	64.6, CH_2_	4.54, s	62.2, CH_2_
13		170.1, C		
14	2.09, s	20.6, CH_3_		
1’				128.5, C
2’			7.68, s	121.1, CH
3’				126.5, C
4’				147.9, C
5’			6.80, d, 7.9	115.9, CH
6’			6.95, d 7.9	123.7, CH
8’				169.1, C
9’			2.05, s	23.6, CH_3_
10’			4.33, d, 6.18	45.2, CH2
2-NH_2_/NH	6.81, brs, NH_2_		7.60, d, 6.1, NH	
11-OH			5.33, br, s	
4’-OH			9.73, br, s	
7’-NH			9.31, s	

Compound **3** was purified as a red yellow powder, and its molecular formula was evidenced to be C_22_H_19_N_3_O_5_ from the quasimolecular ion peak at *m/z* [M + Na]^+^ 428.1217 in its HR-ESI-MS spectrum, which was consistent with ^1^H and ^13^C NMR data (Table 1; Supplementary Figures S13–S19). The ^1^H NMR (DMSO-*d*_6_, 600 MHz) spectrum of **3** exhibited five aromatic protons signals (*δ*_H_ 6.04, 6.36, 7.45, 7.38 and 7.58) and one imino proton resonance (*δ*_H_ 7.60) and one oxygenated methylene signal (*δ*_H_ 4.54 and 5.33), and the ^13^C NMR (DMSO-*d*_6_, 125 MHz) spectrum displayed 11 aromatic carbon resonance lines (*δ*_C_ 96.3, 145.7, 103.3, 149.3, 140.7, 115.6, 127.2, 140.0, 125.39, 133.5, 149.9), one carbonyl carbon signal (*δ*_C_ 179.9) and one methylene carbon (*δ*_C_ 63.2). These data showed almost no difference with exfoliazone described in the literature (Zhang et al., 2015). Apart from these signals of exfoliazone skeleton, there were six aromatic carbon (*δ*_C_ 128.5, 121.1, 126.5, 147.9, 115.9, and 123.5), two aromatic proton (*δ*_H_ 7.68 and 6.80), one carbonyl carbon (*δ*_C_ 169.1), one methyl carbon (*δ*_C_ 23.6), one methyl proton (*δ*_H_ 2.05), one methylene carbon (*δ*_C_ 45.2) one methylene proton (*δ*_H_ 4.33), one imino proton (*δ*_H_ 7.68) and one hydroxyl proton (*δ*_H_ 9.73) as shown in the Table 1. These unassigned data indicated that a subgroup N-(2-hydroxy-5-methyl-phenyl)-acetamide were existed in **3,** which corresponded to be increase in molecular weight of **3** by 164 amu compared to that of exfoliazone. These hypotheses were verified by the key HMBC (Fig. S12) correlations in compound **3**. Thus, the metabolite **3** was identified as a new natural product, named exfoliazone C.

Compound **4** was obtained as a yellow red powder, and its molecular formula C_21_H_22_N_2_O_9_ was deduced from HR-ESI-MS (*m/z* [M + H]^+^ 447.1378, [M + Na]^+^469.1205 calcd for C_21_H_23_N_2_O_9_, 447.1404 and C_21_H_22_N_2_O_9_Na, 469.1223), which was consistent with the ^1^H and ^13^C NMR data (Table 2; Supplementary Figures S20–S26). The ^1^H NMR (DMSO-*d*_6_, 600 MHz) spectrum of **4** exhibited five aromatic protons signals *δ*_H_ 6.45 (1H, s, H-1), 6.44 (1H, s, H-4), 7.54 (1H, d, *J* = 8.4 Hz, H-6), 7.52 (1H, d, *J* = 9.5 Hz, H-7) and 7.76 (1H, s, H-9; one methylene proton *δ*_H_ 5.18 (2H, s, H-11); one methyl proton *δ*_H_ 2.08 (3H, s, H-14) and one imino proton *δ*_H_ 6.96 (1H, d, *J* = 6.7 Hz, NH-8′); and the ^13^C NMR (DMSO-*d*_6_, 125 MHz) spectrum displayed 11 aromatic carbon resonance lines *δ*_C_ 99.6 (CH, C-1), 145.1 (C, C-2), 103.4 (CH, C-4), 148.9 (C, C-4a), 141.7 (C, C-5a), 116.0 (CH, C-6), 129.3 (CH, C-7), 133.5 (C, C-8), 127.5 (CH, C-9), 133.3 (C, C-9a), 148.4(C, C-10a), one carbonyl carbon signal 179.6 (CO, C-2), one methylene carbon signal 64.6(CH_2_, C-11)and one methyl carbon signal 20.6 (CH_3_, C-14). These proton and carbon signals of substructures were very similar to that of metabolite **1** suggesting that **4** might be a derivative of **1**.The residual proton signals *δ*_H_ 4.49 (1H, m, H-1′), 3.37 (1H, m, H-3′), 3.15 (1H, m, H-4′), 4.98 (1H, d, *J* = 5.2 Hz, OH-4′), 3.30 (1H, m, H-5′), 5.06 (1H, d, *J* = 4.3 Hz, OH-5′), 3.34 (1H, m, H-6′), 5.23 (1H, d, *J* = 5.3 Hz, OH-6′), 3.70 (1H, m, H-7), 3.46 (1H, m, H-7′), 4.50 (1H, d, *J* = 5.1 Hz, OH-7′) and carbon signals *δ*_C_ 83.3 (C-1′), 77.0 (C-3′), 70.0 (C-4′), 78.1 (C-5′), 72.2 (C-6′), 60.8 (C-7′) indicated that a hexose were existed in **4**. These substructures were pieced together by the 2D NMR experiments including its ^1^H-^1^H COSY spectrum highlighting the two coupling sequences (11-H to 7-H and 9-H), and its HMBC spectrum (Supplementary Figure S12) indicating the key correlations of H-1 to C-2 and C-3, and H-4 to C-3, C-2 and C-10a, and H-6 to C-5a and C-8, and H-7 to C-11 and C-5a, and H-9 to C-11 and C-5a, and H-11 to C-7 and C-9, and H-14 to C-1, and H-8′ to C-1, C-3, C-1′ and C-6′. Thus, the structure of metabolite **3** was determined as a new glycosylated derivative of **1** and named exfoliazoneglycoside.

**Table 2:** ^1^H and ^13^C NMR data for compound **4** in DMSO-*d*_6_.

No.	*δ*_H_, mult (*J* in Hz)	*δ*_C_,
1	6.45, s	99.6, CH
2		145.1, C
3		179.6, C
4	6.44, s	103.4, CH
4a		148.9, C
5a		141.7, C
6	7.54, d, 8.4	116.1, CH
7	7.52, d, 9.5	129.3, CH
8		133.5, C
9	7.76, s	127.5, CH
9a		133.3, C
10a		148.4, C
11	5.18, s	64.6, CH_2_
13		170.2, C
14	2.09, s	20.6, CH3
1′	4.49, m	83.3, CH
3′	3.37, m	77.0, CH
4′	3.15, m	70.0, CH
5′	3.30, m	78.1, CH
6′	3.34, m	72.2, CH
7′	3.70, m	60.8, CH
	3.46, m	
8′	6.96, d, 6.7	
4′-OH	4.98, d, 5.2	
5′-OH	5.06, d, 4.3	
6′-OH	5.23, d, 5.3	
7′-OH	4.50, d, 5.1	

The known compounds **5**–**10** were identified as exfoliazone (**5**) (Zhang et al., 2015), N-(2-Hydroxy-5-methylphenyl acetamide (**6**) (Suzuki et al., 2006a), 3-acetylamino-4-hydroxybenzaldehyde (**7**) (Suzuki et al., 2006b), 3-acetylamino-4-hydroxybenzonic acid (**8**) (He et al., 2018), viridomycin A (**9**) and viridomycin *F* (**10**) (Omura et al., 1999), by comparing the NMR and MS data with those reported in the literature. It was reported that the known compounds N-(2-Hydroxy-5-methylphenyl) acetamide (**6**), 3s-acetylamino-4-hydroxybenzaldehyde (**7**) and 3-acetylamino-4-hydroxybenzonic acid (**8**) were derived from the precursor 3-amino-4-hydroxybenzoic acid (3,4-AHBA; Gould et al., 1996). The remaining aminophenoxazinones were presumably synthesized from the precursor 3,4-AHBA in the gene cluster, which contained a key tyrosinase-like copper-containing monooxygenase responsible for the C-nitrosation (Noguchi et al., 2010). *Streptomyces* has been reported as pigments producer (Chatragadda et al., 2019b; Zhu et al., 2020). Our results enriched the pigment molecules diversity produced by the *Streptomyces*.

### 3.4. Cytotoxicity of the new metabolites **1**–**4**

*In vitro* toxicity of the compounds **1**–**4** was evaluated against the cell lines using the MTT method (Table 3). The results showed that the metabolites **2**–**4** showed no cytotoxic activities against the human normal liver cell line L-02 with the IC_50_ values of more than 100 μM. These safety performance of **2**–**4** might be an advantage for applying in the food industry. Furthermore, the metabolite **2** showed selective cytotoxic activities toward human ovarian cancer cell line SKOV-3 and gastric cancer cell line MGC-803 with the IC_50_ values of 75.78 and 69.88 μM, respectively, which were lower than those of positive azithromycin with the IC_50_ values of 0.41 and 1.57. The compound **1** exhibited inhibitory activity against the tested human cancer cell lines (human malignant melanoma cell line A375, SKOV-3, breast cancer cell line MDA-231 and MGC-803) with the IC_50_ values of 22.33–67.70 μM. However, it is low toxicity against the normal cell L-02 with the IC_50_ value of 72.01 μM would be a limitation in using as a food pigment.

**Table 3:** Cytotoxic activity (IC_50_ in μM) of the secondary metabolites **1**–**4.**

Metabolites	A375	SKOV-3	MDA-231	MGC-803	L-02
**1**	67.70 ± 4.31	22.33 ± 3.78	55.85 ± 7.47	50.18 ± 7.01	72.01 ± 7.22
**2**	>100	75.78 ± 5.77	>100	69.88 ± 1.94	>100
**3**	>100	>100	>100	>100	>100
**4**	>100	>100	>100	>100	>100
Azithromycin^a^	0.79 ± 0.13	0.41 ± 0.12	4.03 ± 0.43	1.57 ± 0.03	7.34 ± 1.15

a Positive control.

### 3.5. Antibacterial activities of the metabolites

The disc diameters of zone of inhibition (ZOI) values of metabolites (**1**, **2** and **4**) against bacterial pathogens were presented in Table 4; Supplementary Figure S27. The results showed that metabolite **1** possessed strong antibacterial activity against methicillin-resistant *S. aureus* (MRSA) with the ZOI of 15.3 mm, which was equal to that of referenced levofloxacin (ZOI = 15.2 mm). In addition, the metabolite **1** showed moderate antibacterial activities against Gram-positive *S. aureus* (ZOI = 17.2 mm) and *M. tetragenus* (ZOI = 15.mm), which were weaker than those of referenced levofloxacin with the ZOI values of ZOI = 28.9 and 24.8 mm, respectively. And in the MICs test (Table 4), the compound **1** exhibited potential antibacterial activities against *M. tetragenus*, *S. aureus* and MRSA in the MIC tests with the MIC values of 12.5, 12.50, and 25.0 μg/ml, which were comparable to those of positive gentamycin sulfate with the MIC values of 3.13, 3.13, and 6.25 μg/ml, respectively. However, both compounds **2** and **4** did not showed antibacterial activities against the test strains. By comparing the structure of compounds **1** and **2**, the oxhydryl substituent at C-11 in **2** could reduce antibacterial activities against tested bacteria. It indicated that the 11-position hydroxyl group might play a very important role in inhibiting the growth of the Gram-positive bacteria.

**Table 4:** Zone of inhibition (ZOI, mm) and minimum inhibitory concentrations (MICs; μg/ml) of the second metabolites **1**, **2**, and **4** against the tested bacteria.

Metabolites	*Escherichia coli*		*Micrococcus tetragenus*		*Staphylococcus aureus*		MRSA	
	ZOI	MIC	ZOI	MIC	ZOI	MIC	ZOI	MIC
**1**	–^b^	>100	15.3 ± 0.2	12.5	17.2 ± 0.2	12.5	15.3 ± 0.1	25.0
**2**	–^b^	>100	–^b^	50.0	–^b^	>100	–^b^	>100
**4**	–^b^	>100	–^b^	>100	–^b^	>100	–^b^	>100
Levofloxacin^a^	33.0 ± 1.0	3.13	24.8 ± 0.6	3.13	28.9 ± 0.8	3.13	15.2 ± 0.1	6.25

a Positive control 30 μg/disc.

b No inhibited.

## 4. Conclusion

Here, the metabolites of the strain *S. tanashiensis* BYF-112 associated with the termite (*O. formosanus*) were investigated. The results showed that iron elements had a great influence on the color of fermentation broth and metabolites profile of BYF-112. Total of four new natural aminophenoxazinones pigments, along with two rarely green pigments were purified and identified from the fermentation broth of BYF-112 for the first time. Both the yellow and green metabolites showed good stability under heat, light and metal conditions. And the new compounds showed weak or almost no cytotoxicity to the normal cell line L-02. Especially, the metabolite **1** presented strong antibacterial activity against the MRSA, and moderate activities against Gram-positive *M. tetragenus* and *S. aureus*. Therefore, these new natural compounds showed stability and safety as food pigments to a certain extent. In summary, the termite-associated *S. tanashiensis* BYF-112 can be considered a promising new source of bioactive natural pigments that can be used for industrial applications in the future.

## Data availability statement

The original contributions presented in the study are included in the article/Supplementary material, further inquiries can be directed to the corresponding author.

## Author contributions

YZ designed and supervised the study. SZ, JW, ZJ, LZ, TS, and XL performed the experiments and analyzed the data. SZ wrote the manuscript together with CY. All authors contributed to the article and approved the submitted version.

## Funding

This work was supported by the National Natural Science Foundation of China (NSFC; 32102272, 32011540382, and 32270015) and Science Funds for Distinguished Young Scholars of Anhui Province (2108085J18).

## Conflict of interest

The authors declare that they have no known competing financial interests or personal relationships that could have appeared to influence the work reported in this paper.

## Publisher’s note

All claims expressed in this article are solely those of the authors and do not necessarily represent those of their affiliated organizations, or those of the publisher, the editors and the reviewers. Any product that may be evaluated in this article, or claim that may be made by its manufacturer, is not guaranteed or endorsed by the publisher.

## Supplementary material

The Supplementary material for this article can be found online at: https://www.frontiersin.org/articles/10.3389/fmicb.2022.1110811/full#supplementary-material
