# Supplementary material for: Pigments of aminophenoxazinones and viridomycins produced by termite-associated Streptomyces tanashiensis BYF-112
Source: Front Microbiol. 2023 Jan 16;13:1110811. doi: 10.3389/fmicb.2022.1110811 (PMC9884962; doi:10.3389/fmicb.2022.1110811)
Supplement: Supplementary file 1 [file Data_Sheet_1.doc]

**Pigments of Aminophenoxazinones and Viridomycins Produced by Termite-associated *Streptomyces tanashiensis* BYF-112**

Shuxiang Zhang^a^, Jun Wu^a^, Zhou Jiang^a^, Le Zhang^a^，Tao Song^a^, Xinhua Liu^b^, Caiping Yin^a^, Yinglao Zhang^a*^

^a^ School of Life Sciences, Anhui Agricultural University, Hefei, China

^b^ School of Pharmacy, Anhui Medical University, Hefei, China

^*^Corresponding author: Prof. Dr. Yinglao Zhang

E-mail: [zhangyl@ahau.edu.cn](mailto:zhangyl@ahau.edu.cn) Tel.: +86-551-6578-6129

**Supporting information description**

**Figure S1**．The fermentation broth analysis of BYF-112 cultured in YMS and YMS treated with Iron.

**Figure S2.** ^1^H NMR spectrum of compound **1** at 600 MHz in DMSO-*d*_6_.

**Figure S3.** ^13^C NMR spectrum of compound **1** at 150 MHz in DMSO-*d*_6_.

**Figure S4.** ESI mass spectra of compound **1**.

**Figure S5.** ^1^H NMR spectrum of compound **2** at 600 MHz in DMSO-*d*_6_.

**Figure S6.** ^13^C NMR spectrum of compound **2** at 150 MHz in DMSO-*d*_6_.

**Figure S7.** DEPT spectrum of compound **2** at 150 MHz in DMSO-*d*_6_.

**Figure S8.** ^1^H-^1^H COSY spectrum of compound **2** at at 600 MHz in DMSO-*d*_6_.

**Figure S9.** HMQC spectrum of compound **2** at 600 MHz in in DMSO-*d*_6_.

**Figure S10.** HMBC spectrum of compound **2** at 600 MHz in DMSO-*d*_6_.

**Figure S11.** ESI mass spectra of compound **2**.

**Figure S12．**Key HMBC and ^1^H-^1^H COSY correlations of new metabolites **2**-**4**.

**Figure S13.** ^1^H NMR spectrum of compound **3** at 600 MHz in DMSO-*d*_6_.

**Figure S14.** ^13^C NMR spectrum of compound **3** at 150 MHz in DMSO-*d*_6_.

**Figure S15.** DEPT spectrum of compound **3** at 150 MHz in DMSO-*d*_6_.

**Figure S16.** ^1^H-^1^H COSY spectrum of compound **3** at at 600 MHz in DMSO-*d*_6_.

**Figure S17.** HSQC spectrum of compound **3** at 600 MHz in in DMSO-*d*_6_.

**Figure S18.** HMBC spectrum of compound **3** at 600 MHz in DMSO-*d*_6_.

**Figure S19.** ESI mass spectra of compound **3**.

**Figure S20.** ^1^H NMR spectrum of compound **4** at 600 MHz in DMSO-*d*_6_.

**Figure S21.** ^13^C NMR spectrum of compound **4** at 150 MHz in DMSO-*d*_6_.

**Figure S22.** DEPT spectrum of compound **4** at 150 MHz in DMSO-*d*_6_.

**Figure S23.** ^1^H-^1^H COSY spectrum of compound **4** at at 600 MHz in DMSO-*d*_6_.

**Figure S24.** HMQC spectrum of compound **4** at 600 MHz in in DMSO-*d*_6_.

**Figure S25.** HMBC spectrum of compound **4** at 600 MHz in DMSO-*d*_6_.

**Figure S26.** ESI mass spectra of compound **4**.

**Figure S27.** The antimicrobial activities of metabolite **1.**


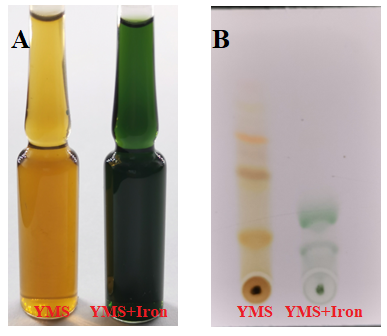


**Figure S1．**The fermentation broth analysis of BYF-112 cultured in YMS and YMS treated with Iron. A: The colours of the fermentation broth of BYF-112. B: TLC analysis of the EtOAc extracts of BYF-112.

**Figure S2**. ^1^H NMR spectrum of compound **1** at 600 MHz in DMSO-*d*_6_.

**Figure S3**.^13^C NMR spectrum of compound **1** at 150 MHz in DMSO-*d*_6_.

**Figure S4**. ESI mass spectra of compound **1**.

**Figure S5**. ^1^H NMR spectrum of compound **2** at 600 MHz in DMSO-*d*_6_.

**Figure S6**. ^13^C NMR spectrum of compound **2** at 150 MHz in DMSO-*d*_6_.

**Figure S7**. DEPT spectrum of compound **2** at 150 MHz in DMSO-*d*_6_.

**Figure S8**. ^1^H-^1^H COSY spectrum of compound **2** at at 600 MHz in DMSO-*d*_6_.


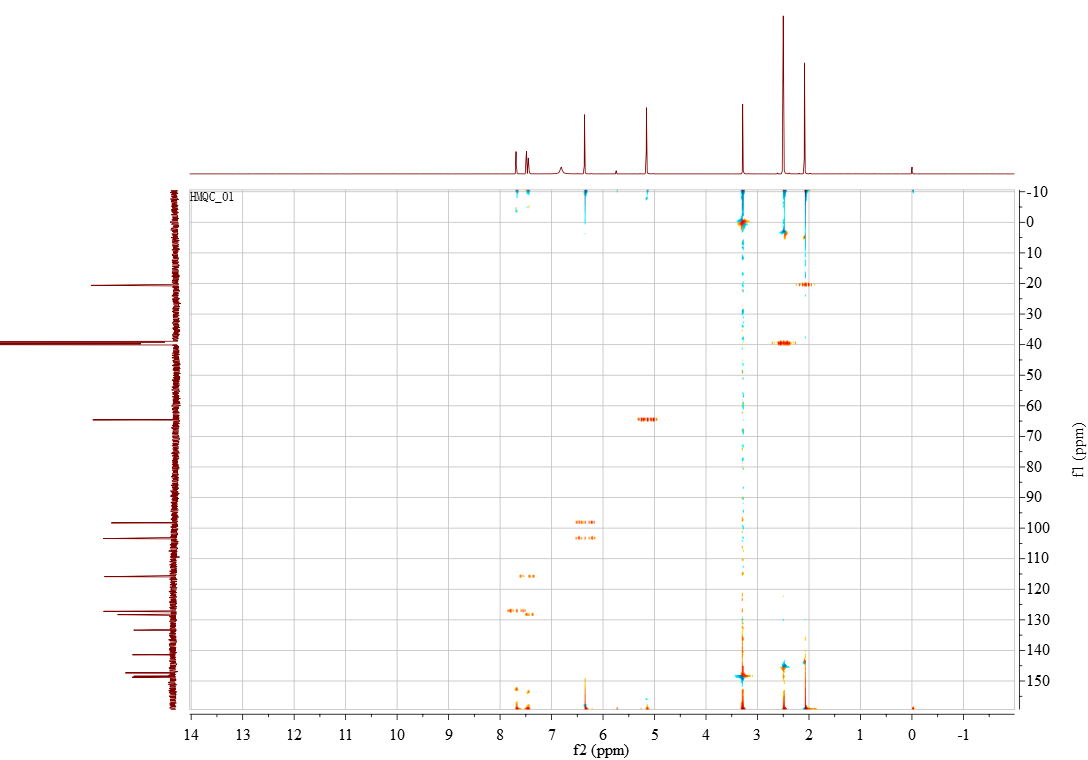


**Figure S9**. HMQC spectrum of compound **2** at 600 MHz in in DMSO-*d*_6_.


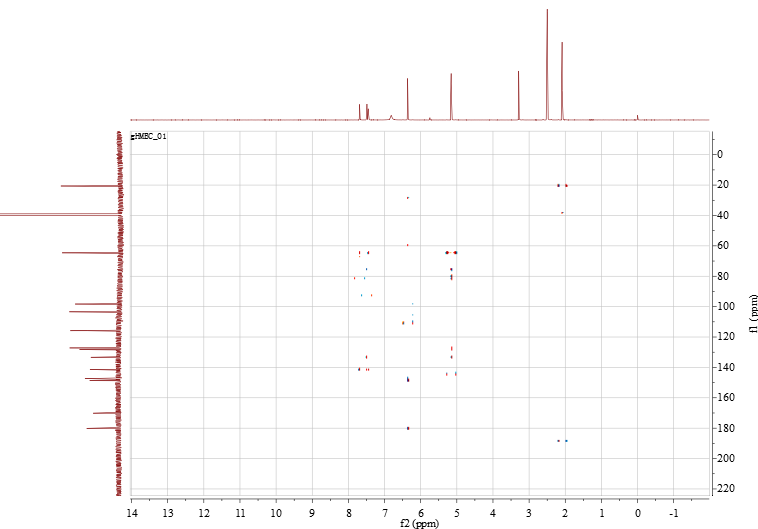


**Figure S10**. HMBC spectrum of compound **2** at 600 MHz in DMSO-*d*_6_.


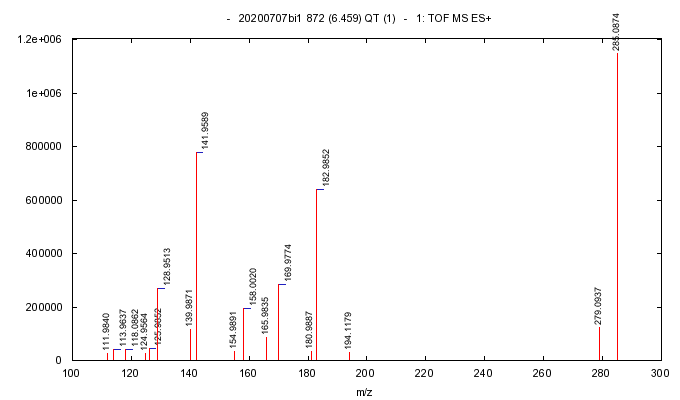


**Figure S11**. ESI mass spectra of compound **2**.

**
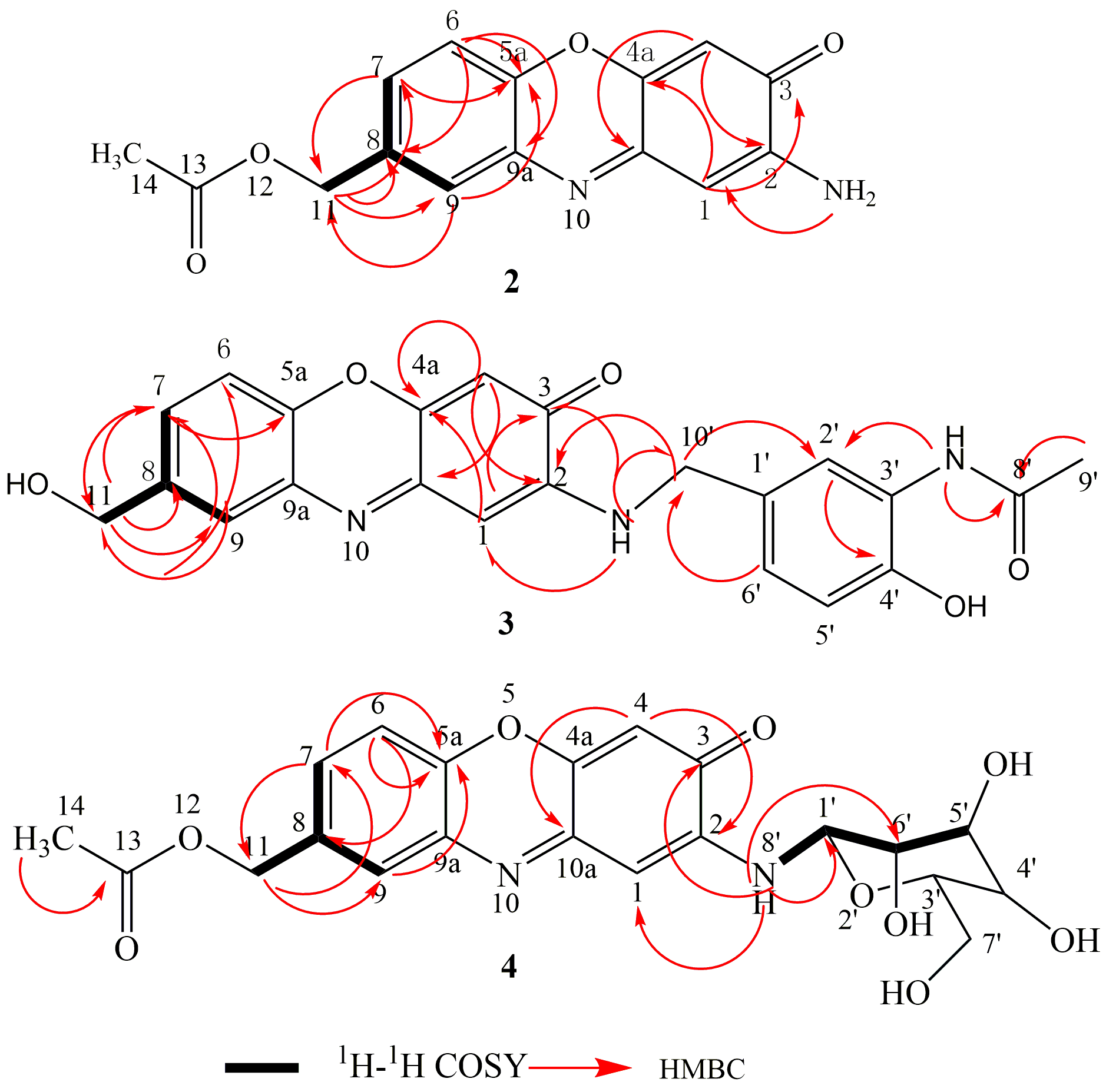
**.

**Figure S12** Key HMBC and ^1^H-^1^H COSY correlations of new metabolites **2**-**4**.


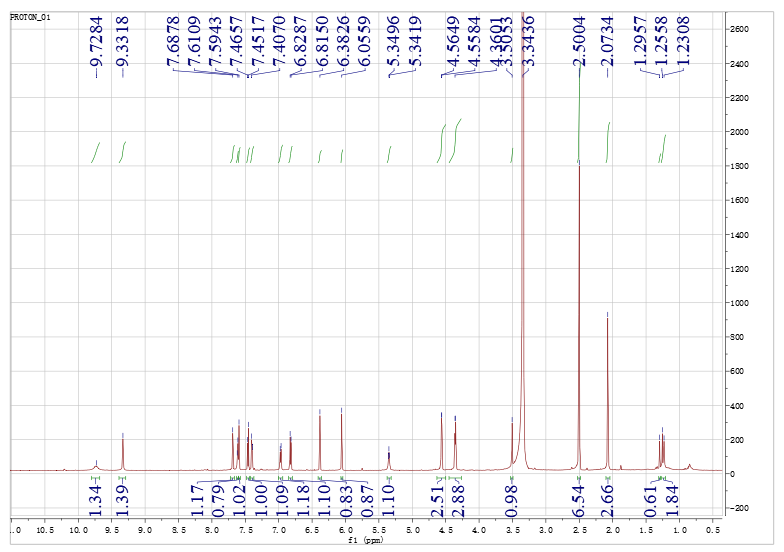


**Figure S13**. ^1^H NMR spectrum of compound **2** at 600 MHz in DMSO-*d*_6_.


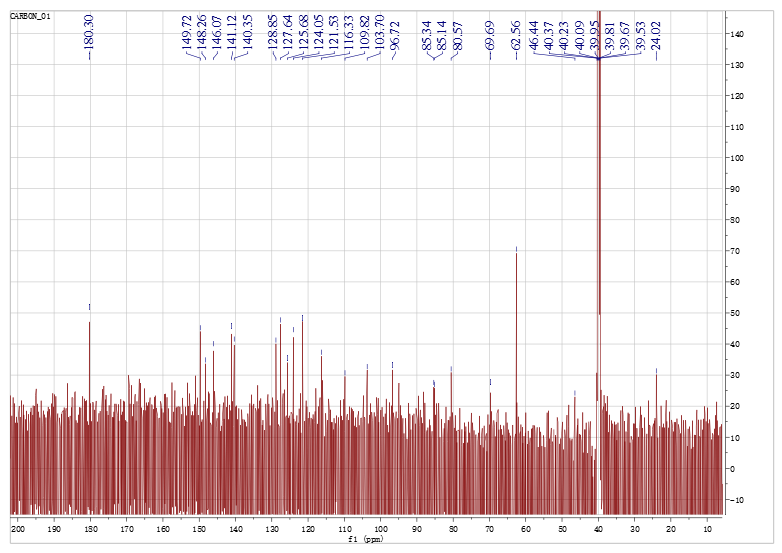


**Figure S14**. ^13^C NMR spectrum of compound **3** at 150 MHz in DMSO-*d*_6_.

**Figure S15**. DEPT spectrum of compound **3** at 150 MHz in DMSO-*d*_6_.


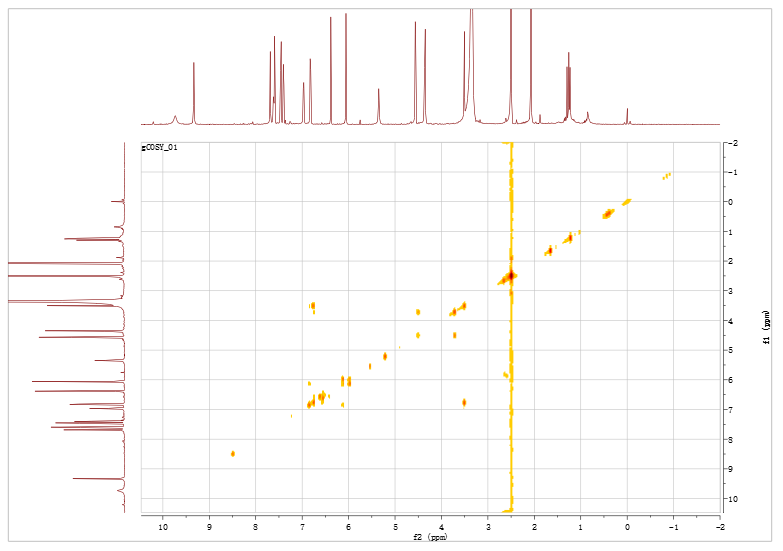


**Figure S16**. ^1^H-^1^H COSY spectrum of compound **3** at at 600 MHz in DMSO-*d*_6_.


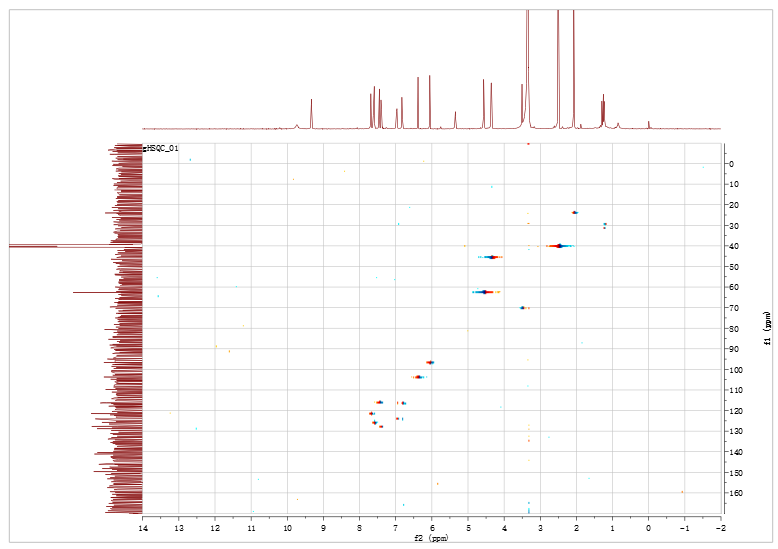


**Figure S17**. HSQC spectrum of compound **3** at 600 MHz in in DMSO-*d*_6_.


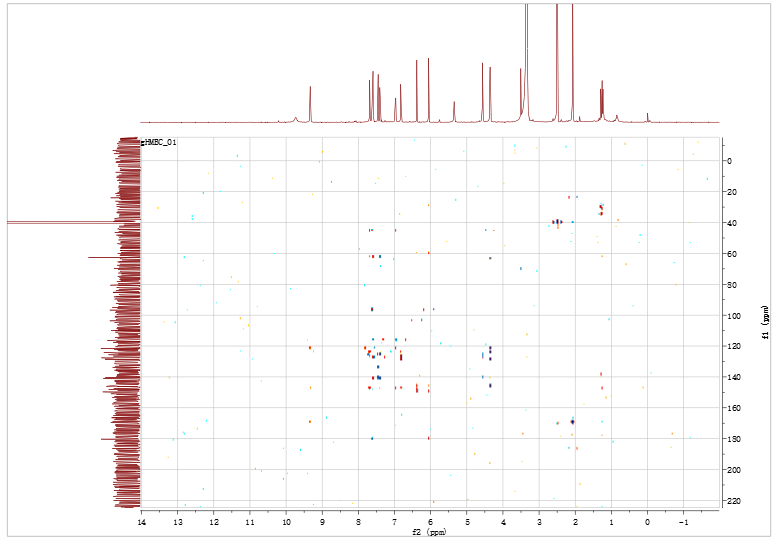


**Figure S18**. HMBC spectrum of compound **3** at 600 MHz in DMSO-*d*_6_.

**Figure S19**. ESI mass spectra of compound **3**.

**Figure S20**. ^1^H NMR spectrum of compound **4** at 600 MHz in DMSO-*d*_6_.

**Figure S21**. ^13^C NMR spectrum of compound **4** at 150 MHz in DMSO-*d*_6_.

**Figure S22**. DEPT spectrum of compound **4** at 150 MHz in DMSO-*d*_6_.


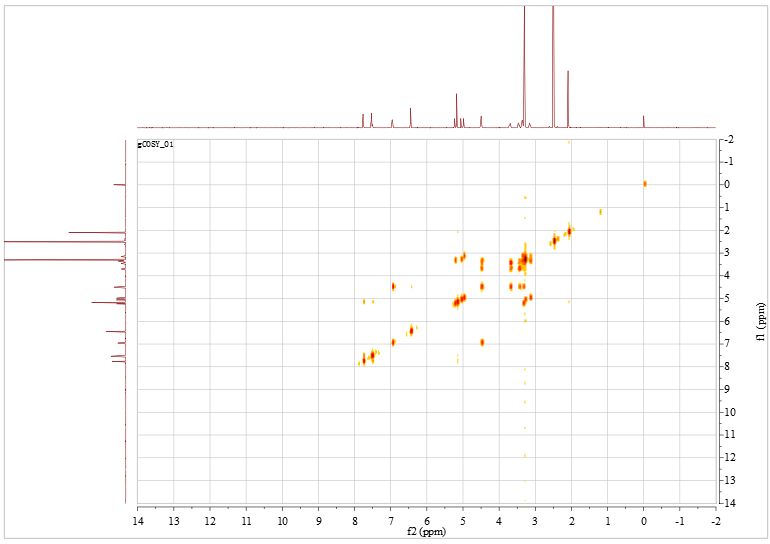


**Figure S23**. ^1^H-^1^H COSY spectrum of compound **4** at at 600 MHz in DMSO-*d*_6_.


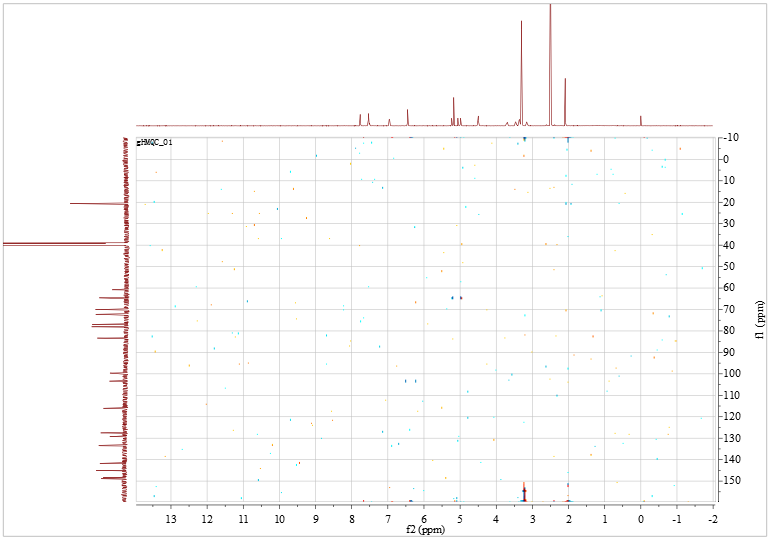


**Figure S24**. HSQC spectrum of compound **4** at 600 MHz in in DMSO-*d*_6_.


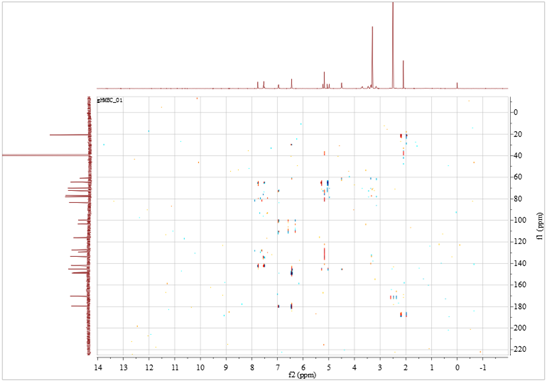


**Figure S25**. HMBC spectrum of compound **4** at 600 MHz in DMSO-*d*_6_.

**Figure S26**. ESI mass spectra of compound **4**.


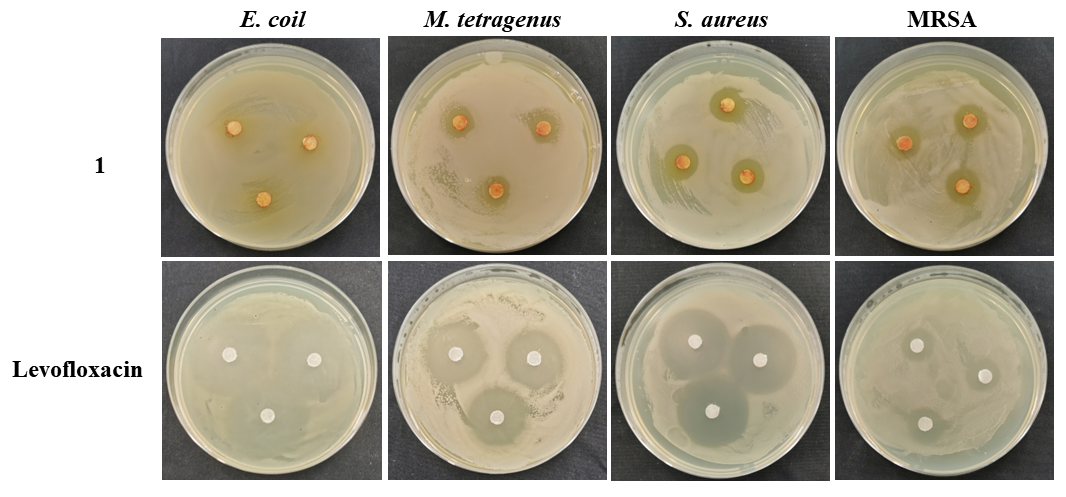


**Figure S27.** The antimicrobial activities of metabolite **1.**
